# Pathogenesis of Human Immunodeficiency Virus-*Mycobacterium tuberculosis* Co-Infection

**DOI:** 10.3390/jcm9113575

**Published:** 2020-11-06

**Authors:** Kevin Wong, James Nguyen, Lillie Blair, Marina Banjanin, Bunraj Grewal, Shane Bowman, Hailey Boyd, Grant Gerstner, Hyun Jun Cho, David Panfilov, Cho Ki Tam, Delaney Aguilar, Vishwanath Venketaraman

**Affiliations:** 1 College of Osteopathic Medicine of the Pacific-NorthWest, Western University of Health Sciences, Lebanon, OR 97355, USA; kevin.wong@westernu.edu (K.W.); james.nguyen@westernu.edu (J.N.); lillie.blair@westernu.edu (L.B.); marina.banjanin@westernu.edu (M.B.); bunraj.grewal@westernu.edu (B.G.); shane.bowman@westernu.edu (S.B.); hailey.boyd@westernu.edu (H.B.); grant.gerstner@westernu.edu (G.G.); hyun.cho@westernu.edu (H.J.C.); david.panfilov@westernu.edu (D.P.); choki.tam@westernu.edu (C.K.T.); delaney.aguilar@westernu.edu (D.A.); 2 College of Osteopathic Medicine of the Pacific, Western University of Health Sciences, Pomona, CA 91766, USA

**Keywords:** HIV, tuberculosis, glutathione, T cell exhaustion, granuloma formation, TNF-α, tumor necrosis factor

## Abstract

Given that infection with *Mycobacterium tuberculosis* (*Mtb*) is the leading cause of death amongst individuals living with HIV, understanding the complex mechanisms by which *Mtb* exacerbates HIV infection may lead to improved treatment options or adjuvant therapies. While it is well-understood how HIV compromises the immune system and leaves the host vulnerable to opportunistic infections such as *Mtb*, less is known about the interplay of disease once active *Mtb* is established. This review explores how glutathione (GSH) depletion, T cell exhaustion, granuloma formation, and TNF-α upregulation, as a result of *Mtb* infection, leads to an increase in HIV disease severity. This review also examines the difficulties of treating coinfected patients and suggests further research on the clinical use of GSH supplementation.

## 1. Introduction

Infection with human immunodeficiency virus (HIV) compromises the body’s immune system, increasing the likelihood of contracting opportunistic infections. According to the World Health Organization (WHO), in 2019, 770,000 people died from HIV. An estimated third of these deaths were attributable to tuberculosis (TB) [1]. HIV and TB are considered a syndemic, defined as “the convergence of two or more diseases that act synergistically to magnify the burden of disease” [2]. Today, TB remains the leading infectious cause of death in people with HIV, who are 15–22 times more likely to contract TB than people without [1].

Currently, the Center for Disease Control and Prevention (CDC) recommends that all patients with active or latent TB be screened for HIV because HIV is a well-known activator of latent TB [3,4]. Approximately one quarter of the global population is estimated to be latently infected with *Mycobacterium tuberculosis* (*Mtb*) also referred to as latent tuberculosis (LTBI) [1]. An individual with LTBI has a *Mtb* infection, but the bacteria remains dormant and inactive within the host’s body with no clinical symptoms [5]. In HIV negative patients, a latent TB infection has a 10% chance of progressing into an active infection over the course of a lifetime. However, this risk is greatly augmented in HIV positive patients and it incrementally increases as immune function declines [6,7,8]. Individuals with HIV and TB also have the potential to influence the health of HIV negative individuals because the reactivation of LTBI makes *Mtb* highly infectious [9]. Additionally, HIV has been linked to several outbreaks of TB globally, especially in sub-Saharan Africa [10].

Both HIV and TB are managed with antiretroviral therapy (ART) and anti-TB therapy, respectively, but despite these potential treatment options, very few patients receive treatment worldwide [11,12]. In 2018, 44% of all patients with HIV associated TB did not receive care [13].

Much is known about the syndemic relationship of HIV and TB, yet the specifics and mechanisms behind how these two diseases interact have not been entirely addressed. This review will explore how *Mtb* worsens HIV infection. The objective of this review is to provide an insight into the interaction between *Mtb* and HIV to help overcome the difficulties in co-treatment of HIV and TB.

## 2. Pathogenesis of HIV

HIV is known to gradually deplete native and memory CD4+ T cells and ultimately result in acquired immunodeficiency syndrome (AIDS). HIV infection has been demonstrated to inhibit T helper 1 (Th1) cell activity and increase T helper 2 cell (Th2) activity [14]. This aberration is often referred to as a Th1/Th2 cell switch [14,15], a phenomenon that Romangnani and colleagues suggest is due to preferential HIV replication within Th2-like cells over Th1 cells that stimulate their proliferation [16]. It is thought that higher levels of CD8+ T cells is a compensatory response for falling CD4+ levels [17]. The shift from Th1 to Th2 is responsible for the observed decreased expression of IL-2 and IFN-γ and increased expression of cytokines such as IL-4, IL-5, and IL-13 [15]. Viral proteins gp120 and gp160, a set of surface proteins on HIV, seem critical to this shift, which also induces Th2 production of IL-4, a stimulator of IgE production and inhibitor of Th1 differentiation [18,19]. The depletion of CD4+ T cells severely compromises the host’s immune response, leaving the individual vulnerable to opportunistic infections such as TB [20]. In an acute HIV infection, type 1 interferons play a critical role in response to HIV infection of T cells by inducing a pro-inflammatory state that slows viral replication [21]. Late-stage HIV infection similarly involves both type 1 interferons and interferon stimulated gene 15 (ISG15) protein as part of the antiviral response [17]. There is evidence that ISG15 protein, a protein released by type 1 interferons, inhibits HIV replication by disrupting the ubiquitination of Gag required for HIV replication [22].

More recently, it was discovered that CD4+ T cell permissiveness enables HIV destruction of CD4+ T cells [23]. HIV recruit′s caspase 3 to initiate cell apoptosis in active permissive CD4+ T cells. HIV also destroys inactive nonpermissive CD4+ T cells by recruitment of caspase 1 to induce pyroptosis, an apoptotic mechanism accompanied with markedly increased levels of inflammation. This mechanism may help explain the nonspecific symptoms such as acute fever, diarrhea, malaise, fatigue, and weight loss, that occur shortly after contracting HIV. Caspase 1 release of pro-inflammatory cytokine IL-1β seems to underlie the HIV-mediated chronic inflammation conducive to viral replication [23,24].

Dendritic cells (DCs) are professional antigen presenting cells that, among other roles, are responsible for initial HIV uptake in anogenital mucosa and subsequent transport to lymph nodes and T cells [25]. HIV manipulates surface receptor CCR5 for entry into DCs, a receptor that is highly expressed in CD11C+ DCs, a DC subgroup found exclusively in the human genital region [26]. HIV exploits the DCs antigen presentation abilities to present virion fractions to CD4+ T cells. To ensure access, HIV also facilitates DC maturation [25].

Many viral infections result in mitochondrial dysfunction, which contributes to an accumulation of reactive oxygen species (ROS). NADPH oxidases and cytochrome P450 2E1 (CYP2E1) serve as the major sources of ROS in hepatitis C, influenza, and HIV [17]. For HIV specifically, envelope protein Gp120 enhances the damage caused by ROS accumulation. In astrocytes, Gp120 upregulates expression of CYP2E1 and increased ROS [27]. ROS can lead to tissue damage and chronic levels can lead to the development of spontaneous tumors [28]. ROS production during HIV infections may also help initiate cell apoptosis by causing telomeric DNA damage [29]. HIV-induced ROS also leads to chronic inflammation that triggers the immune response and may increase the likelihood of developing comorbidities similar to that of ageing. For instance, an HIV infected person, like a diabetic, is two times more likely to develop cardiovascular disease, three times more likely to have a fracture, and is at an increased risk of kidney disease [30].

## 3. Pathogenesis of Tuberculosis

Inhaled as infected droplets, *Mtb* initiates infection by infiltrating both alveolar macrophages and epithelial cells but preferentially multiply in alveolar macrophages [31,32,33]. Upon being engulfed by the macrophages, *Mtb* containing phagosomes prevents fusion with proton-ATPase-containing lysosomes and coats itself with large amounts of the antacid 1-TbAd, effectively evading maximal acidification of the phagosome and thereby protecting *Mtb* from macrophage degradation [34,35]. Upon sensing the phagosomal internal environment, *Mtb* reprograms its own transcriptome to increase iron scavenging and switch from aerobic to anaerobic respiration [36]. Roughly 48 h after initial infection, *Mtb* leverages ESAT-6 secretion system 1 (ESX-1) to secrete virulence factors ESAT-6 and CFP-10 that mediates its escape from the phagosome to enter the cytosol of the macrophage where it can replicate more rapidly [37]. In murine models, *Mtb* redirects alveolar macrophages from the alveolar airspaces into the lung interstitium in an independent process from initial macrophage recruitment that similarly requires ESX-1 [33]. The presence of *Mtb* triggers local inflammatory signals to continue recruitment of macrophages, neutrophils, among other immune cells, mediating the innate immune response that contributes to the formation of the granuloma. During early granuloma formation, the host’s adaptive immune system attempts to respond by sending DCs to the granuloma to engulf bacteria for antigen processing, which will then be presented in the lymph nodes and spleen. *Mtb*, however, can inhibit MHC II antigen presentation without reducing surface MHC II expression in murine DCs, suppressing a robust host adaptive response [38].

Granulomas are a key feature of tuberculosis pathology. Ramakrishnan broadly defines a granuloma as “a compact, organized aggregate of mature macrophages that arises in response to a persistent stimulus” [39]. As the granuloma matures, recruited macrophages transform into an aggregation of “foamy” macrophages that express DEC-205, resembling dendritic cells, with interspersed aggregates of CD4+ cells and some CD8+ cells [40]. The macrophage characteristic foamy appearance is a result of lipid accumulation within their cytosol in response to receptors TR4 and TLR2 binding to *Mtb* cell wall [41]. While granulomas are typically regarded as static structures that isolate an infection, zebrafish models have demonstrated that *Mtb* likely spreads from the primary granuloma via infected alveolar macrophages and establishes secondary granulomas throughout the lungs [42].

As *Mtb* infection progresses, granulomas can undergo necrosis, creating a cheese-like substance called caseum that is extremely difficult to penetrate with antibiotics [43]. The intracellular conditions of lipid-laden foamy macrophages provide both nutrition and an environment that is suitable for *Mtb* bacilli survival [44]. The observation that the foamy macrophage intracellular lipid profile resembles the extracellular contents of caseum suggests that foamy macrophages help create caseum, contributing to TB pathology [45]. The exact trigger for granuloma necrosis is unknown, but Orme and Basaraba suggests neutrophils are likely key to this process [40]. Indeed, *Mtb* appears to both successfully recruit and evade neutrophils in the granulomas of mice subjects, utilizing neutrophilic oxidative burst and proteolytic enzymes like elastase to create a focus of necrotic tissue, suitable for *Mtb* growth [46]. In mice, the walls of the growing granuloma eventually encroach upon alveolar walls through neutrophilic infiltration but the initiation of extensive destruction of these cell walls marks the irreversible process of liquefactive necrosis and cavitation [47]. Interestingly, HIV+ *Mtb* patients consistently have lower frequencies of cavitation suggesting CD4+ T cells likely mediate this process [2]. While the hypoxic environment of caseum inhibits *Mtb* proliferation, cavitation expansion into the lung’s airways creates an oxygen-rich environment that favors *Mtb* growth and dissemination from the primary granuloma [43,48].

## 4. Glutathione Depletion

**Figure 1:**
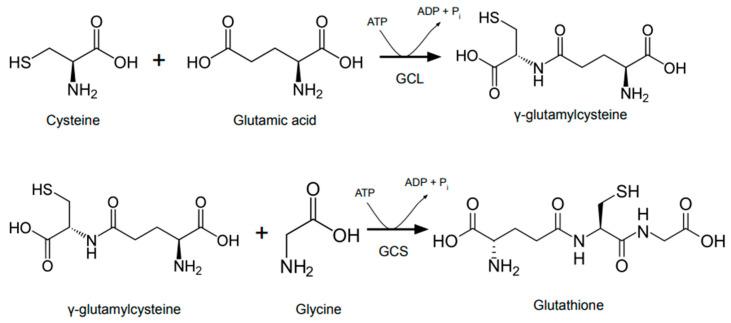
Two step *de novo* synthesis of glutathione. First step is catalyzed by glutamate-cysteine ligase (GCL) and is considered the rate limiting step. Glutamyl cysteinyl glycine synthase (GCS) catalyzes the second step. Both steps require ATP.

Glutathione (GSH) is an antioxidant tripeptide produced by all cells though hepatic production is the primary determinant of in plasma GSH levels [49]. In the extracellular environment, GSH concentrations are low [49,50]. Intracellular GSH levels are a function of *de novo* synthesis and GSH recycling. Glutathione *de novo* synthesis is a two-step process with glutamylcysteinylglycine synthase (GCS) catalyzing the rate liming first step (Figure 1). The cell also uses glutathione reductase (GSR) with NADPH input to recycle oxidized glutathione (GSSG) back into its reduced form [51]. GSH, catalyzed by glutathione peroxidase (GPx), provides protection from intracellular oxidants and pathogens by balancing redox reactions [52]. For this reason, GSH:GSSG ratio, along with NADPH:NADP ratio and thioredoxin_red_/thioredoxin_ox_ ratio, can broadly characterize a cell’s antioxidant capacity [50]. Several therapies, such as *N*-acetylcysteine supplementation, have attempted boosting intracellular GSH levels in hopes of maximizing antioxidant capacity [53]. GSH supplementation in a group of HIV+ patients with low CD4+ counts restored cytokine balance and redox homeostasis in these patients [54].

While it was established early on that diminishing plasma levels of GSH is progressive and associated with asymptomatic HIV patients, it was unclear whether the viral activity simply depleted GSH reserves through consumption or inhibited *de novo* synthesis [55,56]. Through complex mechanisms, antiretroviral therapies and HIV virulence factors reportedly increase ROS that consume GSH while HIV-1 Tat, a protein necessary for efficient transcription, seems to downregulate enzymes in *de novo* synthesis [57,58]. Interestingly, one study analyzing the cytokine profile in different types of HIV+ elite controllers off antiretroviral therapy suggest that the relationship between inflammation and viremia is not straightforward; HIV can impose chronic inflammation, characterized by increased soluble CD163 levels, a marker for macrophage activation and increased arterial wall uptake of ^18^fluorine-2-deoxy-D-glucose associated with atherosclerotic inflammation, that is independent of both antiretroviral therapy and viral replication [59]. Chronic HIV-mediated proinflammatory state can lend itself to the development of insulin resistance and frailty [60,61].

The exact mechanism linking GSH and *Mtb* infections is complex and not yet fully understood but may be associated with inflamed, necrotic tissue. Granulomas are the primary structures that create a pro-inflammatory state in the host. While pro-inflammation TNF-α is crucial for granuloma integrity, TNF-α dysregulation can be detrimental. Excessive TNF-α can induce macrophage necrosis mediated through mitochondrial ROS, RIP1 and RIP3 kinases [62]. As *Mtb* proliferates and the infection progresses, granulomas can caseate and eventually undergo liquefaction to form a cavity [63]. Exposure to the granuloma and *Mtb* itself can cause a dysregulated cytokine and chemokine profile that promotes a proinflammatory state that can be sustained by both bacterial burden and necrotic tissue [64]. Increased immune cell activity and liberated intracellular contents from necrotic cells both release ROS into the extracellular space that promotes proinflammatory responses and oxidative stress, likely consuming GSH (Table 1) [65,66].

GSH supplementation seems to be crucial in boosting the function of different immune cells. In an ex vivo study with blood samples from HIV+ *Mtb* infected patients, it was found that liposomal GSH treatment could restore Th1-related cytokine response previously impaired by HIV infection [67]. GSH treatment increased proinflammatory cytokines IL-1β, IL-12, IFN-γ, and TNF-α while decreasing anti-inflammatory cytokines TGF-β and IL-10 and free radicals. In other studies, GSH enhanced the function of macrophages and natural killer cells against *Mtb* infection [50,68]. In HIV patients, Herzenberg and colleagues suggested GSH deficiency predicted worse survival rates that could be offset by replenishing GSH levels through oral *N*-acetylcysteine supplementation. In accordance to prior in vitro studies, their results supported claims tying low GSH levels with decrease in cell survival, T cell function modification, accelerated HIV replication, increased NF-kB activation and increased sensitivity to TNF-α-induced cell death [69].

In summary, independently, HIV and *Mtb* infections are responsible for triggering a chronic, inflammatory state [24]. Therefore, GSH, as a free radical scavenger, plays an integral role in responding to both HIV and *Mtb* infections. In response to these infections, GSH depletion is likely caused by increased antioxidant function, as well as downregulation of enzymes in biosynthetic pathways [70]. Given that HIV protein Tat can inhibit GSH *de novo* synthesis, L-GSH supplementation could be an optimal treatment that bypasses this inhibition, restoring host immune function against infections including *Mtb* [55,67,70]. This could be particularly promising in HIV and *Mtb* coinfections where each infectious process builds upon each other to create a negative synergistic state that considerably impairs the immune system. In fact, GSH supplementation in one in vitro study with HIV+ *Mtb* infected immune cells helped overcome HIV-mediated T-helper cell impairment, limiting *Mtb* growth [71]. However, further research needs to be directed towards understanding whether in vivo GSH supplementation is suitable and efficacious as an adjuvant therapy to HIV+ *Mtb* infected patients.

## 5. Tuberculous Granuloma

Not all granulomas are similar in content; as HIV progresses, the characteristics of TB granulomas evolve [72]. Studies indicate that peripheral CD4 (pCD4) T cells play a significant role in granuloma formation. Patients with greater than 300 pCD4 T cells/mm^3^ developed well-formed granulomas while patients with 200–300 pCD4 T cells/mm^3^ developed a mix of well-formed and poorly formed granulomas [73]. Patients with under 50 pCD4 T cells/mm^3^ do not form well-organized granulomas [74]. These patients also expressed reduced CD4 and CD8 T cells, and reduced IFN-γ production leading to further *Mtb* abundance [75]. Patients with HIV and low pCD4 T cell counts are therefore associated with underdeveloped granuloma formation [76].

Although limited, studies explain that tuberculous granulomas can change the microenvironment to enhance HIV replication [77]. HIV replicates preferentially in activated CD4+ T cells and macrophages and due to their major role in tuberculous granuloma formation, they are likely a replication hotspot for HIV. In their study, Hoshino and colleagues, highlight the importance of macrophage activation in the presence of inflammatory cytokines in stimulating HIV replication by upregulating nuclear factor kappa-light-chain-enhancer of activated B cells (NF-κB) to de-repress the HIV long terminal repeats (HIV-LTR). They also revealed how cell-to-cell contact between neutrophils and macrophages de-represses the HIV-LTR through a reverberating interaction that increases the viral load within granulomatous lesions [78].

Garrait and colleagues revealed that the microenvironment within the granulomatous lesion has elevated levels of cytokines. This study showed that pro-inflammatory cytokines, most notably TNF-α and IL-6, are upregulated while IL-10 is simultaneously downregulated. In short, by increasing pro-inflammatory cytokines and decreasing anti-inflammatory cytokines, HIV replication is stimulated within macrophages and T cells [79]. The literature contains some controversy over the role of TNF-α and disease progression. Noronha and colleagues [80] demonstrated that the microenvironment of TB granulomas within HIV infected individuals contains significantly lower levels of TNF-α. This supports the findings of The Food and Drug Administration (FDA) that TNF-α antagonists have been linked to reactivation of LTBI. This is likely due to the crucial role of TNF-α, and other cytokines, in host response against *Mtb.* Thus, by using TNF-α antagonists, *Mtb* may replicate unopposed [81]. Although the data is conflicting, TNF-α has been proven to be a key cytokine within the granulomas of HIV/TB infected patients.

## 6. T Cell Exhaustion in HIV and *Mtb* Infections

**Figure 2:**
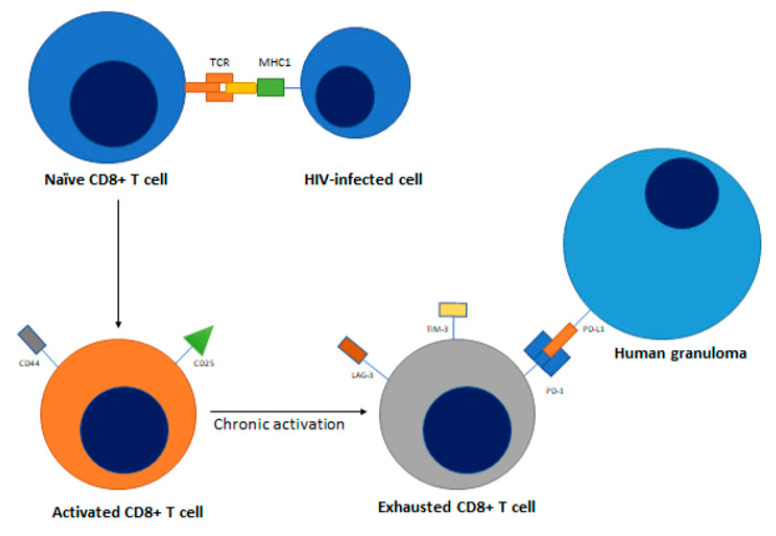
Naïve CD8+ T cells undergo exhaustion after chronic activation by HIV antigen mediated MHC I presentation, with downregulation of activation markers like CD25 and CD44, and upregulation of exhaustion markers like PD-1, LAG-3, and TIM-3. Its activation is also hindered by the PD-1/PD-L1 interaction with human granulomas.

As part of the adaptive immune system, T cells play a vital role in mitigating pathogen proliferation chiefly through coordinating the host’s immune response and inducing apoptosis of infected cells. The initial response to HIV infection follows an archetypal response. In an acute infection, T cell activation requires two signals to occur simultaneously: T cell receptor (TCR) recognition of processed antigens on the surfaces of antigen-presenting cells (APCs) and co-stimulation of T cell ligand CD28 with APC CD80/86. Upon activation, T cells secrete proinflammatory cytokines such as IFN-γ and TNF-α to combat the pathogen. Following pathogen clearance, approximately 90–95% of activated T cells undergo apoptosis while the remaining differentiate into memory T cells via action by IL-7 and IL-15 [82]. Immune checkpoint receptors, found on the surface of T cells, regulate immune response by extinguishing immune cell activity upon clearance of a pathogen or tumor cell. Important immune checkpoint receptors include lymphocyte activation gene protein (LAG3), T-cell immunoglobulin domain and mucin domain-containing protein 3 (TIM3), cytotoxic T lymphocyte antigen-4 (CTLA-4) and programmed cell death receptor 1 (PD-1). If these immune checkpoint receptors become upregulated, proliferating T cells can become unresponsive to stimulation, entering a state called T cell exhaustion, eventually succumbing to apoptosis (Figure 2) [83,84,85], in which checkpoint ligands like PD-1 interacts with PD-L1 expressed by human granulomas [86] leads to reduced T cell cytotoxicity, proliferation, and activation [87].

HIV-mediated T cell exhaustion is well documented. As HIV transitions to chronic infection, CD8+ T cells downregulate CD127 and CD122, receptors for IL-7 and IL-15, respectively, while upregulating activation markers, such as CD25 and CD44. With these losses, the proliferation of activated T cells declines significantly [88]. Additionally, exhausted T cells, particularly effector T cells, exhibit dysregulated cytokine profiles, favoring secretion of anti-inflammatory cytokine IL-10 over pro-inflammatory cytokines such as IL-2, IFN-γ and TNF-α [82,83,89]. While exhaustion was first observed in CD8+ T cells, CD4+ T cells also undergo similar effects. Typically, CD4+ T cells secrete IL-2 and IFN-γ but chronic viral infections can significantly alter their cytokine profile to resemble those of their CD8+ counterparts with upregulation of programmed cell death protein 1 (PD-1) and downregulation of IL-2 and IFN-γ [90,91,92].

Additionally, in response to TCR stimulation, CD8+ cells require both calcineurin-dependent transcription factor and nuclear factor of T cell activation (NFAT) to promote inflammatory cytokines [93]. Upregulation of thymocyte selection associated high mobility group box (TOX) is central to creating sustained exhaustion because it increases PD-1 expression on CD8+ T cells [94,95]. Hence, reversing the T cell exhaustion through blocking immune checkpoints such as PD-1 has been targeted to enhance host response to infectious diseases [96]. Like HIV infections, chronic *Mtb* infections may lead to T cell exhaustion. During primary infection of *Mtb* in the lungs and hilar lymph nodes, most acute infections become latent by forming granulomas [97]. When the lungs are inoculated, T cell progenitors secrete IFN-γ to activate macrophages. In turn, these macrophages secrete cytokines essential for granuloma formation (IL-1, TNF-α) and T cell differentiation into Th1 and Th17 subsets (IL-12, IL-6). Both types of T helper cells secrete cytokines that support the integrity of the granuloma (IL-17, IL-2, TNF-α). However, in chronic infection, these cytokines are downregulated in T cells. Subthreshold levels of these cytokines permit breakdown of the granuloma, opening the possibility of activating a latent TB infection into its miliary form. This is further corroborated by DiNardo and colleagues’ ex vivo study that found an increase in anti-inflammatory cytokines (IL-4 and IL-10) and a decrease in pro-inflammatory cytokines (IFN-γ) correlating with reactivation of latent TB, seen also in HIV disease progression [98]. Using similar cytokines involved in HIV infection, *Mtb* also exacerbates T cell exhaustion through related mechanisms. In response to chronic *Mtb* infection, TIM3 and PD-1 of T cells are overexpressed, causing an inadequate response to *Mtb* infection [99]. Jayaraman and colleagues showed that the upregulation of TIM3 correlated with decreased secretions of IL-2, TNF-α, but increased IL-10 [85]. As described previously, dysregulation of cytokine expression, most notably TNF-α, caused by chronic *Mtb* infection, can induce a shift from a pro-inflammatory state to an anti-inflammatory state that compromises the integrity of the granuloma, leaving the host vulnerable to disease progression (Table 1).

**Figure 3:**
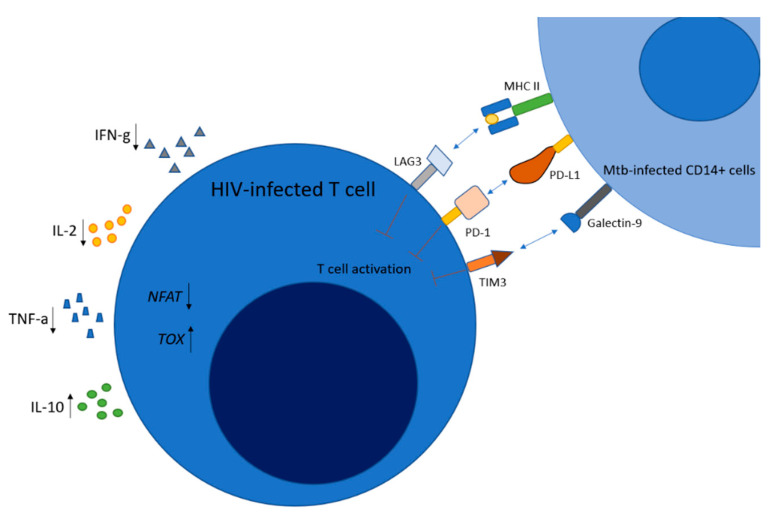
*Mtb-infected CD14+ cells mediate T cell exhaustion.* Ligands from *Mtb*-infected CD14+ cells inhibit T cell activation, thereby decreasing intracellular nuclear factor of T cell activation (NFAT), but increasing TOX proteins and inflammatory cytokines. IFN-γ and IL-2 secretion decrease, while the secretion of the immunosuppressive cytokine, IL-10, increases.

Upregulated in T cell exhaustion, PD-1, receptor for PDL-1 expression on HIV-specific CD8+ T cells correlates with viral load [100]. Additionally, HIV infection reduces the number of *Mtb*-specific Th1 CD4+ T cells and IL-2-producing CD4+ and CD8+ T cells with increased PD-1 expression on *Mtb*-specific CD4+ and CD8+ T cells [101]. During the early stages of HIV infection, CD4+ T cells mitigate viral replication by differentiating into Th1 helper T cells. However, as the viral load of HIV increases, CD4+ T cell secretion of IFN-γ reduces, further diminishing activation of Th1 helper T cells. This finding has been corroborated by Lalvani and colleagues [90], showing an inverse relationship between plasma levels of *Mtb*-specific polyfunctional CD4+ T cells that secrete IFN-γ and IL-2 (Figure 3), and HIV-1 viral load, suggesting that HIV-1 infection and *Mtb* work synergistically to suppress CD4+ T cells. Lastly, *Mtb* increases expression of LAG3 antigens in CD4+ T cells, weakening Th1 response against *Mtb*-infected macrophages [102]. Fenwick and colleagues [84] speculate that this mechanism underlying increased morbidity seen in HIV+ *Mtb* patients is *Mtb*-mediated impaired Th1 activation compounded with HIV-mediated decreases in overall CD4+ cells create an inflammatory state that favors HIV viral replication.

While previous studies focused on how HIV increases vulnerability to *Mtb* infections, there is considerably less literature studying how *Mtb* infections exacerbate HIV infections. Recently, it was observed in sub-Saharan Africa how *Mtb* immunosuppression may promote HIV-1 progression to AIDS [103]. Furthermore, ex vivo studies demonstrated that both patients with latent and active *Mtb* infections had similar increases in whole blood inflammatory cytokines IL-6, IFN-α2, IL-18, and IFN-γ associated with increased susceptibility of HIV-1 transmission between isolated CD4+ cells [104].

Lastly, recent in vitro studies on IFN-1 and IL-10/STAT3 dependent tunneling nanotube formation in macrophages have highlighted how *Mtb* supports HIV vectoring between CD4+ cells (Table 1) [105,106]. As mentioned earlier, exhausted T cells favor secretion of IL-10 over pro-inflammatory signals [82,83,85,89]. The findings of IL-10 mediated tunneling provide a possible mechanism for viral spread between CD4+ cells, while also suggesting a key role of IL-10 in an exhausted state, especially as the HIV disease state progresses [85,103,104,105,106]. While the overall mechanism of HIV exacerbation from *Mtb* is not yet completely elucidated, fully understanding T cell exhaustion in the context of HIV+ *Mtb* infections and developing therapies aimed at preventing T cell exhaustion may prove useful in slowing HIV progression in HIV+ *Mtb* patients.

## 7. TNF-α Upregulation

TNF-α is a regulatory cytokine produced by monocytes [107]. In HIV infections, TNF-α increases HIV gene expression through receptor-mediated induction of the HIV promoter region, playing an important role in the HIV disease progression. TNF-α has been shown to be upregulated in both HIV and *Mtb* infections, especially in HIV infections when specifically signaled by transactivation of *tat* [107,108]. Tat is a regulatory protein that increases efficiency of viral transcription, and when taken up by host cells, send stimulatory or inhibitory signals for cell proliferation dependent on cell type [109,110]. Therefore, Tat can suppress the immune system through inhibition of antigen induced T cell proliferation [108].

TNF-α increases HIV expression in T lymphocytes and monocytes by activating NF-kB transcription factor through peroxidative mediation [107]. Tat also indirectly modulates NF-kB activity by augmenting TNF-α response to increased oxidative stress Tat protein increases TNF-α activity directly by inhibiting Mn-dependent superoxide dismutase (Mn-SOD) which is associated with decreased GSH expression [84]. Inhibition of Mn-SOD permits increases superoxide anion concentrations, consuming GSH reserves and thus increasing oxidative stress that feeds back to amplify TNF-α activity. The cellular redox state is the primary determinant of tat-TNF-α-NF-kB modulation [109].

Increased levels of serum TNF-α is associated with AIDS patients. It has been proposed that TNF-α has an inductive effect on HIV replication with increased binding of NF-κB to the HIV long terminal repeat. The maintenance of constitutive production of HIV expression is likely due to the endogenous production of TNF-α (Table 1) [110]. For each individual, selective inhibition of TNF-α, IL-2, IL-1ꞵ, INF-γ were shown to partially decrease levels of *Mtb*-induced HIV replication. Simultaneous inhibition of TNF-α and the same other cytokines dramatically reduced the levels of *Mtb*-induced HIV expression [111]. This suggests that HIV expression is caused by autocrine mediation via TNF-α [112].

TNF-α is also necessary for the development and maintenance of granulomas inside epithelioid cells which kill ingested bacteria such as *Mtb*. Loss of function of TNF-α can then be concluded to lead to opportunistic *Mtb* infections. Cultures infected with *Mtb* secreted higher levels of TNF-α and other cytokines [111]. Anti-TNF-α treatments prevent the maturation of granulomas which enabled unencumbered *Mtb* replication [112]. Pretreatment of cells with anti-TNF-α antiserum greatly diminished the capacity of TNF-α to upregulate HIV expression via targeting HIV reverse transcriptase [110].

## 8. Difficulties in Co-Treatment of HIV and Tuberculosis

### 8.1. Drug-Drug Interactions and Co-Toxicities

Drug-to-drug interactions are a concern in the co-treatment of *Mtb* and HIV. Acquired resistance against rifampin has been increasing amongst HIV patients [113]. Rifampin, however, remains one of the most important anti-TB drugs because it shortens the length of treatment time. Subtherapeutic plasma concentrations of anti-TB drug isoniazid (INH) can also contribute to acquired rifampin resistance (ARR) [114]. Even in HIV-uninfected individuals, suboptimal INH concentrations increase the risk of developing ARR by five-fold. Similarly, intermittent dosing of both rifampin and INH rather than consistent dosing, may lead to the development of ARR in HIV+ patients [115]. Notably, patients who are HIV+ are also at risk for malabsorption of rifampin, again leaving this patient population at greater risk for developing ARR [116]. The WHO currently recommends initiating antitubercular therapy with a two-month period of concomitant dosing of isoniazid, rifampicin, pyrazinamide and ethambutol (HRZE) followed by a four-month period of isoniazid and rifampicin only, ideally with daily dosing [117]. The WHO also recommends HIV+ *Mtb* patients to follow these same guidelines alongside antiretroviral therapy [118]. In patients infected with isoniazid and rifampicin-resistant strains, clofazimine in combination with fluoroquinolones is preferred to decrease duration of treatment [119,120]. Nix-TB is an ongoing phase 3 trial for a novel drug combination BPaL which consists of bedaquiline, pretomanid and linezolid for the treatment of coinfection of HIV and multidrug resistant *Mtb* [121]. In aerobic conditions, pretomanid inhibits molecular components *Mtb* requires for cell wall synthesis. However, in anaerobic conditions, pretomanid releases nitric oxide that kills the bacteria from within [122]. Further research will need to elucidate the complex interplay between HIV and *Mtb* to develop future effective therapies [123].

**Figure 4:**
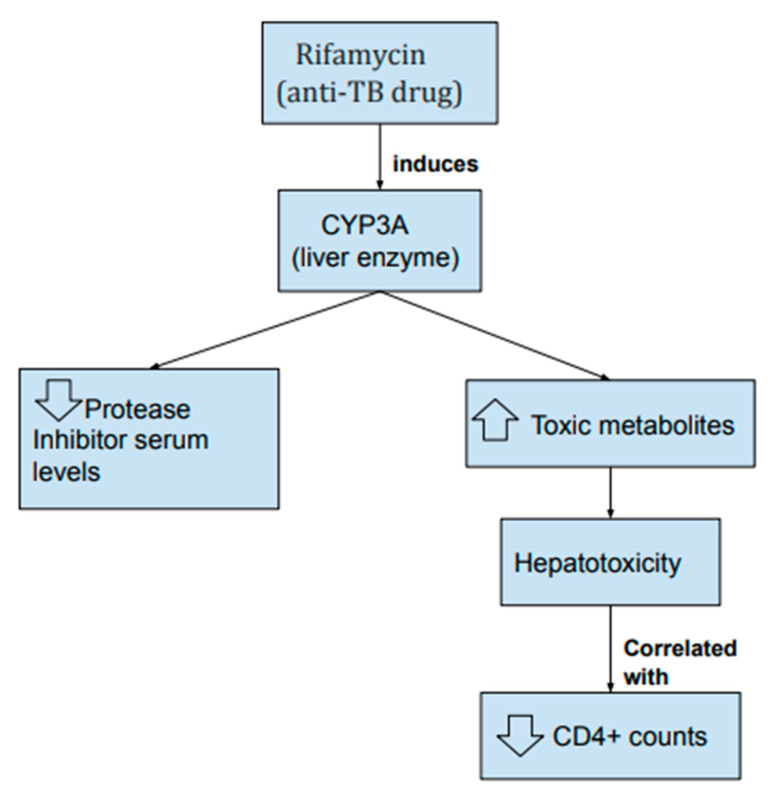
HIV anti-retroviral drugs inhibit metabolism of Rifamycin, an anti-TB drug. The increased levels of Rifamycin lead to induction of liver enzyme CYP3A, which then increases the metabolization of drugs such as nelfinavir, a protease inhibitor used in antiretroviral therapy (ART) against HIV. This decreases the serum level of drugs such as nelfinavir. The induction of CYP3A also leads to the production of toxic metabolites that causes hepatotoxicity, which has been correlated with decreased CD4 + counts.

Rifampin interacts with antiretroviral therapies as well, particularly non-nucleoside reverse-transcriptase inhibitors (NNRTI) and protease inhibitor (PI) nelfinavir [124]. Rifamycin induces liver enzyme CYP3A (Figure 4), therefore substrates of this enzyme, such as nelfinavir, will be metabolized more rapidly (Table 2) [125]. Efavirenz-based HAART is the recommended therapy when treating co-infected patients who are on rifampin, despite decreased serum levels and unknown pharmacokinetics of the drug-drug interactions. Triple NRTIs are not recommended with rifampin because the combination is associated with inferior viral suppression [124]. Despite the risk of drug-to-drug interactions, cure rates for HIV related *Mtb* hover around 95% with the use of first-line drugs, and drug monitoring may only be necessary for patients with inadequate responses in early therapy [126].

Due to the high mortality rate of *Mtb* infection in HIV patients, it is important to start aggressive drug therapy. Unfortunately, one of the issues with concurrent treatment of *Mtb* and HIV is co-toxicities, especially hepatotoxicity [126]. Antimycobacterial and antiretroviral drugs (HIV therapy) share metabolic pathways which likely underlie the co-toxicities seen in concurrent treatment [127]. Development of hepatotoxicity correlates with decreases in CD4+ counts (Figure 4), suggesting immunologic contribution to hepatotoxicity in HIV patients. Rifamycin, a class of anti-TB drugs, have been demonstrated to increase the risk of hepatotoxicity in HIV patients when co-administered with saquinavir/ritonavir (Table 2) [128]. Saquinavir/ritonavir inhibits the breakdown of rifampin, as well as increases generation of toxic metabolites through rifampin mediated induction of CYP3A4 [129]. When dealing with concurrent therapies for *Mtb* and HIV, monitoring patients is key to improving patient outcomes because high risk populations of TB are at risk of higher mortality associated with drug toxicity [130]. Due to the extensive overlap in toxicities in both regimens, guidelines typically suggest delaying ART for some months until TB medications are managed and stabilized [126].

### 8.2. IRIS

Patients upon initiation of ART will experience progressive restoration of immune response against infectious and noninfectious antigens, which predisposes them to developing immune reconstitution inflammatory syndrome (IRIS) [131]. Shelburne and colleagues define IRIS as “paradoxical deterioration in clinical status attributable to the recovery of the immune system” [132]. ART improves HIV markers, such as lower HIV viral load and higher CD4+ T cell count, at the expense of inflammatory responses [133]. IRIS differentiates itself from opportunistic infections by the “unmasking” of an already present but silent infection with an abnormal and excessive immune response. With recovery of immune function, the host can finally mount a robust and frequently exaggerated inflammatory response [131]. TB is a common pathogen associated with IRIS, with a proposed incidence rate of 8–43% [125,134,135]. Some of the most common clinical manifestations of TB-IRIS are fever, lymphadenopathy, pulmonary infiltrate progression, respiratory distress, hepatosplenomegaly, and diarrhea [127,134,136]. Less frequently, TB-IRIS can present with neurological illness such as meningitis and/or brain tuberculomas triggered by inflammation of the central nervous system [135].

There are two types of TB-IRIS: paradoxical and unmasking [127]. A patient with paradoxical TB-IRIS is diagnosed with tuberculosis prior to starting HIV treatment and often has already initiated anti-tubercular treatment (ATT) before ART with improved tuberculosis symptomatology [137]. These patients typically initially improve on ATT followed by a short period of deterioration upon initiating ART [121]. In contrast, the “unmasking” type of TB-IRIS occurs when an individual with no prior TB diagnosis begins experiencing TB-IRIS symptoms after initiation of ART [135]. Paradoxical TB-IRIS is better understood with more extensive research than the unmasking version [138]. While paradoxical TB-IRIS presents with a wide range of manifestations such as respiratory, neurological, or abdominal symptoms, two thirds of unmasking TB-IRIS presents with lung involvement [138,139]. Some of the risk factors for developing paradoxical TB-IRIS are younger age, male gender, and anemia, as well as *Mtb* burden and disseminated disease [138,140]. Unmasking TB-IRIS is often hard to distinguish from paradoxical [138]. It could be of benefit to improve understanding of unmaking TB-IRIS, especially since the patients that develop it do not have prior TB diagnoses and thereby evade monitoring for TB-IRIS when ART is initiated.

The WHO currently recommends all co-infected individuals initiate antiretroviral therapy regardless of their CD4+ count, despite the greater risk of TB-IRIS associated with earlier initiation of ART [141,142]. This recommendation was based on the Cambodian early versus late introduction of antiretrovirals (CAMELIA) trial where there was a demonstrated decrease in mortality for patients that started ART within two weeks of initiating ATT compared to patients started on ART within eight weeks. To enroll into the CAMELIA study, subjects needed a CD4+ T cell < 200 cells/mm^3^ [142]. However, Abdool and colleagues [143] argue that these benefits only occur at much lower CD4+ T cell count cutoff: patients with a <50 cells/mm^3^ had improved mortality and lower risk of developing AIDS that was not seen in those with a CD4+ T cell count >50cells/mm^3^. If ART is initiated soon after the start of antimycobacterial medication for *Mtb,* then the chance of TB-IRIS developing is increased as the *Mtb* load will be greater [134]. Campbell and Dyson postulate that antimycobacterial medications result in antigens that may lead to an “excessive” inflammatory response [144]. Early ART initiation is also associated with greater rates of ART discontinuation because of adverse side effects (Table 2). The greatest benefit of early ART is the shorter period of immunodeficiency seen in co-infected patients; a time period associated with greater risks of mortality [141]. Regardless, initiation of integrated treatment of HIV and *Mtb* requires close physician monitoring during the first couple of weeks of treatment when IRIS is most likely to occur [142].

### 8.3. Adherence

Concurrent treatment of multidrug-resistant (MDR) TB and antiretroviral therapy has shown to improve survival rates amongst co-infected individuals [145]. Effective treatment however requires high adherence rates and is complicated by high pill burden and adverse side effects. Nonadherence, common in both HIV and TB care, can lead to drug resistance, clinical deterioration, and ongoing transmission [146]. MDR treatment consists of using second-line medications that are typically more toxic and require longer treatment periods, and for these reasons, many individuals discontinue treatment [145]. Some of the more common side effects include gastrointestinal issues, vertigo, hearing disturbances and arthralgia, though most patients will still adhere to the TB regimen once the affecting drug has been discontinued [147]. There is some concern of the additive nature of side effects associated with simultaneous ART and MDR TB treatment that threatens adherence to both medication regimens (Table 2) [145]. Providers need to be aware that HIV infections may cause malabsorption of TB drugs further complicating co-treatment [148]. Treatment outcomes for MDR TB between HIV+ patients on ART and HIV-patients were similar except for HIV+ patients whose CD4+ counts were <100 cells/mm^3^ which significantly increased their mortality rate [145]. Brust and colleagues [145] noted medication adherence was high for HIV+ MDR patients despite a high percentage of adverse side effects reported.

In Martinson and colleagues’ study using rifapentine and isoniazid, 95% patients reported adherence to medication regimen at 12 weeks that subsequently dropped to 60.4% at the three-year mark and further dropped to 43.3% at the four-year mark, with patients reporting an average of 3.3 years of medication adherence [149]. Shorter treatments are desirable because of greater adherence rates when compared to continuous treatment. Theoretically, the remaining unused pills from shorter treatments could be instead given to treat previously untreated patients, increasing the total number of people receiving treatment [149,150]. In this rifapentine and isoniazid trial, Martinson and colleagues [151] also noted that 87.3% of subjects in the continuous treatment group also reported adverse side effects. There is further evidence that regular attendance to routine clinical visits were linked to treatment success when compared to those who missed visits. Notably, there was greater adherence to ART than TB treatment attributed to better patient education and greater support for HIV care [146]. ART also has a lower pill burden with fewer adverse side effects that likely contributes to the greater rates of medication adherence compared to TB treatment. Adverse side effects and complex drug interactions are partially responsible for lower TB adherence, but the stigma associated with TB likely also plays an important role. TB patients frequently report healthcare workers who noticeably avoid direct contact with them, leading to demoralization and mistrust of providers that can result in refusal of treatment [152]. The lack of adherence to TB treatment is of great concern due its highly contagious nature, as well as its high mortality rates of coinfection [150].

## 9. Conclusions

HIV attacks its hosts by weakening their immune system, effectively increasing the likelihood of opportunistic infections. The immune system of HIV patients is weakened paradoxically due to alterations in the immune responses [153]. Specifically, CD4+ T cells are depleted, effectively compromising the patient′s immune system over time [99]. This leaves HIV patients at greater risk to opportunistic infections, such as *Mtb*, the leading cause of death in HIV patients worldwide [13].

Due to the high likelihood of *Mtb* infection in HIV patients, the CDC highly recommends that HIV patients are screened for *Mtb* [3]. *Mtb* infection starts with inhalation of *Mtb* which is then taken up by alveolar macrophages. *Mtb* bacilli which are not destroyed by RNI and ROI produced by the macrophages begin to proliferate and yield various immune factors promoting inflammation. This inflammation recruits immune cells, including CD4+ T cells, therefore consolidating HIV′s primary target of infection [154]. It is this connection between the inflammatory nature of *Mtb* and HIV target cells that brings us to the main concept of this review which argues that *Mtb* exacerbates HIV infection.

There are multiple ways by which *Mtb* can worsen HIV infection including T cell exhaustion, GSH depletion, granuloma formation, and upregulation of TNF-α production. The first mechanism to discuss is T cell exhaustion. Due to HIV attacking and depleting CD4+ T cells there are less CD4+ T cells to release immune factors such as IFN-γ and TNF-α, therefore overworking the remaining T cells, resulting in T cell exhaustion. The culmination of these two factors results in increased activity of *Mtb* [99]. The second mechanism by which this occurs is via GSH depletion. There are two processes occurring in *Mtb* patients infected with HIV which results in decreased GSH production and therefore loss of immune function. *De novo* synthesis of GSH is drastically reduced as decreased expression of GSH synthesis enzymes in macrophages was observed. Additionally, there is an increase in production of proinflammatory cytokines, resulting in increased levels of free radicals [155]. With decreased levels of GSH, patients are unable to handle oxidative stress adequately, effectively increasing the rate of HIV infection.

Upon infection with *Mtb*, granulomas form, which contain amongst multiple immunological factors, infected macrophages. It is also suggested that *Mtb* speeds up HIV infection due to the specific formation of granulomas in HIV+ *Mtb* infected individuals. In the following study it was observed that the granulomas formed in HIV+ individuals were poorly formed and exhibited necrosis. Additionally, a marked decrease in TNF-α concentrations was observed, this leads to the conclusion that granuloma formation was worsened due to low levels of TNF-α. These factors therefore exhibit another reason as to how *Mtb* markedly increased the rate of HIV infection [80].

Due to the low levels of GSH observed in HIV+ and *Mtb* patients one proposed adjunctive treatment for these patients is GSH supplementation. The mechanism for this treatment plan works by increasing levels of Th1 cytokines via elevating the response of IL-12, IL-2, and IFN-γ. This benefits HIV+ patients infected with *Mtb* as it is better able to control the symptoms experienced due to *Mtb* infection [67]. Additionally, it was observed that treatment for the complete removal of *Mtb* was successful with the administration of N-Acetyl Cysteine (NAC) in addition to the traditional antibiotics utilized [156]. No adverse effects from the NAC administration were observed during this study, however it is important to note that the subjects in this study were not co-infected with HIV. With the addition of HIV to the treatment of *Mtb*, the treatment becomes more complex and co-toxicity becomes a problem. It was observed that antiretroviral drugs in combination with anti-tb drugs can result in hepatotoxicity due to the shared pathways between these two drug classes [127]. Additionally, it was observed that treatment for *Mtb* followed by anti-retroviral treatment for HIV patients increased the subject’s chances of contracting Immune reconstitution inflammatory response (IRIS), but ultimately reduced mortality [157].

Understanding the interplay between *Mtb* and HIV infection rates is the first step in deciding the best course of treatment for dually infected patients in order to drastically reduce the mortality rate for HIV patients suffering from *Mtb* infections. GSH supplementation may be a potential avenue for treatment for HIV patients. Future research may look at GSH supplementation for patients suffering from co-infections from both *Mtb* and HIV.

## Figures and Tables

**Table 1:** Summary of *Mtb* factors that can exacerbate HIV infections.

Factors of *Mtb*	Effect on HIV
Glutathione depletion	Increases in oxidative stress in areas of infection increase inflammation and permits proliferation of HIV infected immune cells [54,67,71]
Decrease in pro-inflammatory cytokines in chronic infection due to T cell exhaustion	Decreased pro-inflammatory cytokines result in suboptimal immune responses to viral infection allowing for HIV disease progression [82,83,89,90,91,92].
Excess of TNF-α in acute infection	TNF-α is a major cytokine in granuloma formation that recruits macrophages and T cells forming a replication hot-spot for HIV infected immune cells [97,110,111,112].
Increase in anti-inflammatory cytokines in chronic infection due to T-cell exhaustion	Anti-inflammatory cytokines (mainly IL-10) are associated with increasing tunneling nanotubes which facilitate the transfer of HIV between T cells [103,105].

**Table 2:** Summary of *Mtb* treatment factors that can exacerbate HIV infections.

*Mtb* Treatment	Effect on HIV Treatment
Drug–drug interactions	Rifamycin induces liver enzyme CYP3A which increases metabolism of antiretrovirals, notably nelfinavir. Increased CYP3A activity also results in increased toxic metabolites that cause hepatotoxicity and reduce CD4+ counts [124,125,126,127,128,129,130].
IRIS with the initiation of ART	The initiation of ART increases the likelihood of developing IRIS which increases the rate of non-compliance to the anti-TB and ART regimens [131,133,134,135,136].
Treatment adherence	Individually, non-adherence to HIV or TB treatment regimens are common. Treatment for dually infected patients is associated with a higher rate of non-adherence due to side effects, pill burden, and complex drug interactions [145,146,147,148,149,150,151,152].
